# Distribution of soil selenium in China is potentially controlled by deposition and volatilization?

**DOI:** 10.1038/srep20953

**Published:** 2016-02-17

**Authors:** Guo-Xin Sun, Andrew A. Meharg, Gang Li, Zheng Chen, Lei Yang, Song-Can Chen, Yong-Guan Zhu

**Affiliations:** 1 State Key Laboratory of Urban and Regional Ecology, Research Center for Eco-Environmental Sciences, Chinese Academy of Sciences, Beijing 100085, China; 2 Key Laboratory of Urban Environment and Health, Institute of Urban Environment, Chinese Academy of Sciences, Xiamen 361021, China; 3 Department of Environmental Science, Xi’an Jiaotong-Liverpool University, Ren’ai Road, No.111, Suzhou Industrial Park, Suzhou 215123, China; 4 Institute for Global Food Security, Queen’s University Belfast, David Keir Building, Malone Road, Northern Ireland, BT9 5BN, UK

## Abstract

Elucidating the environmental drivers of selenium (Se) spatial distribution in soils at a continental scale is essential to better understand it’s biogeochemical cycling to improve Se transfer into diets. Through modelling Se biogeochemistry in China we found that deposition and volatilization are key factors controlling distribution in surface soil, rather than bedrock-derived Se (<0.1 mg/kg). Wet deposition associated with the East Asian summer monsoon, and dry deposition associated with the East Asian winter monsoon, are responsible for dominant Se inputs into northwest and southeast China, respectively. In Central China the rate of soil Se volatilization is similar to that of Se deposition, suggesting that Se volatilization offsets it’s deposition, resulting in negligible net Se input in soil. Selenium in surface soil at Central China is roughly equal to low petrogenic Se, which is the main reason for the presence of the Se poor belt. We suggest that both deposition and volatilization of Se could play a key role in Se balance in other terrestrial environments worldwide.

Selenium (Se) is an essential micronutrient for humans^1^,^2^, and most Se is primarily derived from cereals, including through secondary consumption as meat^1^,^2^,^3^. In general, the accumulation of Se in edible part of plants is highly dependent on Se level in soils^2^,^4^. In China, human Se deficiency is associated with chronic bone and cartilage disease (Kashin-Beck disease) and chronic heart disease (Keshan Disease)^5^,^6^. These diseases are found in populations living in the Se-poor belt in Central China, which stretches from northeast (Heilongjiang Province) to southwest (Yunnan Province) (Fig. 1A).

Chinese agricultural regions are divided into two broad catchments according to the 400 mm isohyets, an important geographical boundary for summer monsoon limit (Fig. 1B). The west belonging to semi-arid (200–400 mm) or arid climate (<200 mm), is dominated by grassland and the east belonging to semi-humid (400–800 mm) or humid (>800 mm), dominated by arable farming. The extensive Se-poor belt is located in the eastern farming region and accounts for ~70% of Chinese arable land^7^. Extremely low Se in soils within this general Se-poor belt results in widespread Se deficiency in edible produce, which poses a human health risk for ~0.7 billion inhabitants of this area^7^.

At large geographical scales the spatial distribution of Se in the terrestrial environment is poorly understood^8^. Previous studies mainly focused on the transformation and biogeochemical cycling of Se in soils at micro-scale^7^,^9^. It is generally accepted that on the regional scale soil-forming parent materials or rocks are among the most important factors affecting Se distribution in soils^6^,^8^. However, on the national scale such as in China, it remains elusive as to what are the predominant factors in regulating Se distribution (Fig. 1).

Generally, crystalline bedrock and Quaternary sediments have been identified as the major parent materials for soil^10^. The surface bedrock of earth is covered by 75% sedimentary rock and 25% metamorphic and igneous rocks. In China about 77.3% of total area is covered by sedimentary rocks (Fig. 2). Except for some organic-rich rock such as black shale and volcanic emanations, most types of rock contain relatively low Se, roughly in the similar range of Se with crustal abundance^11^. The average crustal abundance of Se worldwide is estimated to be 0.05–0.09 mg/kg^8^. In China, the average abundance of Se in the earth’s crust is 0.058 mg/kg, a bit lower than that in other parts of the world^12^. The Se concentration of various rock types in China is 0.07 mg/kg in metamorphic rocks, 0.067 mg/kg in igneous rocks, and 0.047 mg/kg in sedimentary rocks^12^. The maps of soil parent materials and distribution of soil Se in China should be matched each other if rock-derived Se from parent materials are the main contributor of soil Se, but they do not (Figs 1A and 2). This must mean that the spatial variability of soil Se distribution is affected by other factors besides low Se in soil-forming parent materials or rocks. The mean Se concentrations in soils in northwestern China (Se-adequate region), the middle part (Se-poor belt), and the southeastern part (Se-adequate region) are 0.19, 0.13 and 0.23 mg/kg, respectively^5^. A comparison between the average Se content in different parts of Chinese soils and the average Se concentration in Chinese earth’s crust (0.058 mg/kg) indicates other factors play an important role in elevating Se concentrations in surface soil.

One source of Se in soil besides parent rocks could be the atmospheric deposition^13^,^14^,^15^,^16^,^17^. Dissolved Se in seawater was taken up by phytoplankton and transformed to gaseous Se compounds in marine environments. Significant concentrations of gaseous Se occur in surface ocean waters and emit to the atmosphere^17^,^18^. Marine derived Se is thought to be an important source of Se to terrestrial ecosystems^14^,^15^,^16^,^18^. Countries closer to the ocean tend to have more sufficient Se supply in soil than those in central Europe^9^. High wet deposition was accompanied by high soil Se in southeast China (Fig. 1B), suggesting wet deposition derived from East Asian monsoon is one of the important controlling factors of Se distribution in China^19^. The deposition in Central China is relatively low and geographically coincides with the Se-poor belt (<0.1 mg/kg). Surface soil Se concentration in this region is similar to or slightly higher than Se level in bedrock, much less than that in Northwest or Southeast China, indicating that Se accumulation in surface soil was limited. Monsoonal deposition is an important Se input^19^ and no major Se output was documented, the Se levels in surface soil of Se-poor belt would be progressively increased rather than similar to petrogenic Se level (<0.1 mg/kg). Mechanisms for formation of the Se poor belt in this region remained elusive. In Northwest China, which beyond the range of the East Asian summer monsoon, wet deposition is much less (<200 mm), even less than 100 mm in some regions such as the Gobi desert^20^. However, the high Se in surface soils was observed (Fig. 1A). Obviously, the elevation of Se in northwest China is not due to transport and deposition from the East Asian summer monsoon.

## Results

### Selenium input from wet deposition

**Figure 1:**
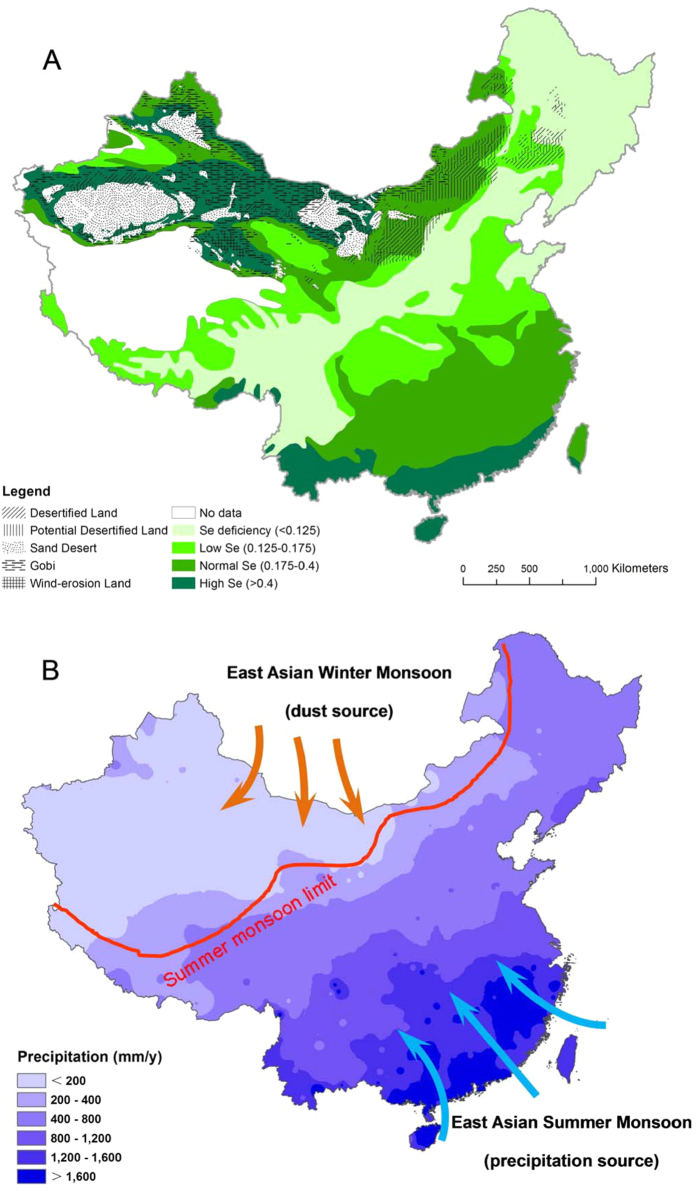
Distribution pattern of soil selenium concentration and desert (**A**), and wet deposition (**B**) in China (China Meteorological Data Sharing Service System, China’s precipitation data of 1971–2000. http://cdc.nmic.cn/home.do). The maps were created using Arc GIS Geographic Information Systems software version 10.2 (Environmental Systems Research Institute Inc, Redlands, Calif).

Annual mean wet deposition decreases gradually from 2,000 mm along the southeastern coast, to 400–800 mm in central China, and less than 200 mm in most of the Northwest (Fig. 1B). According to the deposition gradient derived from Southeast Asia summer monsoon in China, the whole territory can be divided into 3 regions: 1) Southeast humid region (>800 mm); 2) Central dry and humid region (400–800 mm); and 3) Northwestern dry region (<400 mm, beyond summer monsoon) (Fig. 1B). In Southeast humid region, the highest frequency of Se concentration in inland deposition of China is in the range of 0.1–0.2 μg Se/L^8^,^21^, similar to the average value of the deposition samples (0.21 μg Se/L) in UK^22^. Based on the concentrations of Se in rainwater, the Se depositions in the soil at different rainfall were calculated (Table 1).

Following the increase of rainfall (deposition>800 mm) the Se concentration was substantially enhanced in the southeast humid soil region in comparison with Se contents in the bedrock (0.047–0.07 mg/kg). Wet deposition associated with East Asian summer monsoon has been suggested as a major contributor to the distribution of trace elements^23^. The largest natural source of Se to the atmosphere was marine biogenic origin^15^,^16^, up to 60–80% of the atmospheric Se^14^. Gaseous Se derived from oceanic emission is transported through the atmosphere and deposited to terrestrial environments^18^,^24^,^25^. When wet deposition is 2,000 mm annually, the Se input in the surface soil was up to 200–400 μg/m^2^ per year (Table 1).

Central dry and humid regions (400–800 mm) geographically coincide with the Se-poor belt (Fig. 1). The amount of Se deposited by deposition is highly dependent on distance from the ocean. This region is far from ocean Se source and deposition of Se is much less. According to the calculated Se deposition (Table 1), the rate of Se deposition in this region is 40–160 μg/m^2^/yr.

### Selenium input from dry deposition

The Northwestern dry region (<400 mm) exhibit high Se, especially in the arid environment with annual deposition less than 200 mm although no deposition of East Asian summer monsoon occurred. The distribution of Se in this region is surprisingly coincident with the distribution of desertified lands (Fig. 1A). The East Asian monsoon, the largest monsoon system, is an important part of global atmospheric circulation. Unlike African and Indian monsoon, the East Asian monsoon is divided into a warm and wet summer monsoon and a cold and dry winter monsoon, which is a unique and strong winter circulation. Main features of the winter monsoon in East Asia are that very strong high-pressure and low-pressure systems form over the Mongolia and Siberia (High) and the northern Pacific Ocean (Low), respectively. This cold and dry winter monsoon is not only the most powerful winter monsoon worldwide, but also responsible for the mineral dust deposition (Fig. 1B). Asian winter monsoon generates wind patterns in winter that sweep clastic material from the deserts of the Central Asian and deposits it in China. Three areas including deserts in Mongolia, Taklimakan and Badain deserts in western and northern China have been concluded to be the major sources for Asian mineral dust, contributing ~70% of the total dust emitted^26^. The mean rates of dust deposition were 200 g/m/yr at local scale (0–10 km from dust source) and 20 g/m/yr at regional scale (10–1000 km from dust source) respectively, much higher than that (0.4 g/m/yr) at global scale (>1,000 km from dust source)^26^,^27^. Most desertified lands in Northwestern China are located at local scale or regional scale from dust source. Much of the mineral dust raised (~30%) in Asia was redeposited onto the deserts including Gobi, desertified land and potential desertified land (Fig. 1A)^28^.

The total dust production from Chinese deserts is estimated ~ 800 Tg/yr into the atmosphere with a range of 500–1,100 Tg/yr^28^, accounting for half of the global dust production of 1500 Tg/yr^29^ or 1,000–2,000 Tg/yr^30^. Global Se dry deposition has been estimated to be 1.7–2.4 × 10^9^ g Se per year, less than wet deposition of 3.5–10.0 × 10^9^ g Se per year^31^. Northwestern dry region is close to the deserts (the source of deposition in Asia). Aerosol concentration of Se has been demonstrated to be highly enriched relative to average crustal abundances^32^. It is plausible explanation that on most desert margins high Se (>0.4 mg/kg) was accumulated in surface soil due to dry deposition (Fig. 1A). Obviously, dry deposition associated Asian winter monsoon is major contributor for Se elevation in in northwest China.

### Selenium volatilization to decrease soil Se

Selenium volatilization from soil is one of the most important processes of Se biogeochemical cycling in terrestrial environments^33^. Dimethylselenide and dimethyl diselenide are the major volatile Se species emitted to the air from soil^31^. Biological volatilization is regarded as a significant pathway of Se removal in wetlands^31^.

It has been shown that volatilization of Se from soils can be influenced by various factors, including temperature^34^,^35^,^36^, soil moisture^35^,^36^,^37^, and organic C sources^37^,^38^. Many reports have focused on Se volatilization from soils in laboratory incubation experiments^39^,^40^, with amendments of carbon and/or various Se species for enhancement of Se volatilization^41^,^42^. Temperature, Se concentration in soil, and the level of microbial biomass (especially in the rhizosphere) were among the most important environmental factors influencing Se volatilization^43^. These soil factors directly control microbial activity which influences the Se biomethylation and volatilization processes. Selenium volatilization rates vary enormously in the field due to these variables, with very high rates occurring spring and early summer^44^,^45^. For example, the mean rates were 150 and 25 μg Se/m^2^/day in February and October, respectively, in the San Francisco Bay, California, which is heavily contaminated by Se inflow from urban and industrial sources^44^. These factors such as temperature, soil moisture and organic C are quite different from southeast to northwest China, suggesting Se volatilization would be significant difference.

The majority of Se volatilization studies have focused on the Se volatilization in soils and sediments that were either naturally contaminated or amended with Se^37^,^40^,^42^. Few field studies have been conducted to investigate Se volatilization from natural environments^31^,^46^. The data available are too sparse to establish a reliable flux estimate to balance the assumed volatilization from land, limiting our ability to predict volatilized Se fluxes quantitatively. It was reported that the average volatile fluxes of Se in natural wetlands was 0.12 μg/m^2^/day (43.8 μg/m^2^/year). The flux rate of Se volatilization in upland (grassland) was estimated to be on the order of 100–200 μg/m^2^/year^46^,^47^. Given that most regions in China belong to upland soil, we postulate that all soils in humid and semi-humid environment volatilized Se at around this level (100–200 μg/m^2^/year) although many environmental variables (temperature, soil moisture, plant species, organic C levels, etc.) will cause fluctuations in actual Se volatilization.

### Factors affecting Se volatilization and microbial biomass in soil

Volatilization of Se is a product of microbial activity in soil. Soil microorganisms play key roles in Earth’s biogeochemical cycles and global cycling of soil nutrients are strongly influenced by soil microbial communities. Sterilization of seleniferous soil by autoclaving completely eliminates Se volatilization^4^,^48^. Different environment (arid or humid) have different phenotypes of microorganisms, as well as varying populations and, consequently, different microbial activities. Soil microbial biomass and activity are primarily driven by moisture availability, soil nutrients such as nitrogen and organic carbon^49^ and vegetation cover^50^. It has been documented in many experiments that volatilization of Se can be enhanced by different organic amendments and also by many other environmental factors (e.g. soil moisture, soil management, etc.)^35^,^36^,^37^. Volatilization of sulfur, markedly similar to Se, was significant decreased by decreasing soil moisture^51^. Air-drying the soil severely inhibits Se volatilization^52^. Field studies have shown that Se emission rates were much lower at dry sites than in corresponding damp or wet conditions^53^. Global hotspots of abundances of soil microorganisms have been identified in tropical regions and the lowest soil microbial biomass was in arid and semi-arid regions^49^. In comparison with warm and wet tropical and subtropical climates in southeast China, soil microbial biomass would much less in cold and dry arid and semi-arid climate of northwest China. This strongly limits biogeochemical cycling of soil nutrients, including Se volatilization derived by soil microbes.

**Figure 3:**
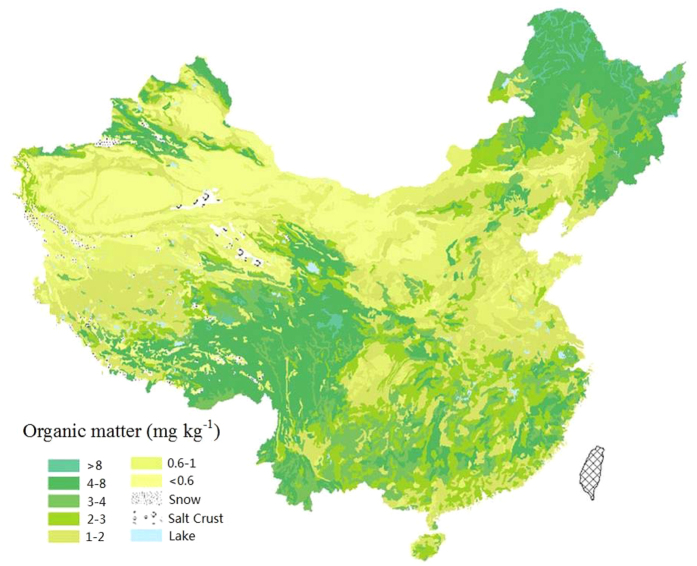
Map of soil organic matter of China (Digital Soil Mapping from Institute of Agricultural Resources and Regional Planning, Chinese Academy of Agricultural Sciences,http://www.caas.ac.cn/datashare/spatialdata/om2ndse.jpg).

Volatilization of Se can be enhanced in the field with an available carbon source. Fierer *et al.*^50^ suggested that soil microbial biomass is strongly correlated to soil organic matter (SOM) content and plant productivity. The positive relationships were reported between soil microbial biomass and the contents of soil organic carbon^50^,^54^. The lowest contents of soil organic matter are in the northwest China (Fig. 3)^55^, which is considered to be one of the limiting resource for soil microorganisms.

Temperature as one of the most important environmental factors influencing Se volatilization^43^, is another key factor limiting soil microbial population. Selenium volatilization is temperature-dependent and positively correlated with soil temperature^56^. In field measurements, volatilization of Se was reported to fluctuate seasonally, with greater rate in the spring and summer than that in the fall and winter, which correlated with soil temperature. The annual average temperature in northwest of China (<−5^o^ C) is much low than in southeast regions (>15^o^ C)^57^, which significantly repress the growth of soil microbes and Se volatilization. Poor plant cover is a factor affecting microbial abundance in northwest China as well. Soil from beneath plants generally have greater microbial population and diversity than soils associated with bare areas (Fuller, 1974), which tend to have greater microbial activity for Se volatilization. Furthermore, in dry environment, bioavailability of Se is less due to the lack of available moisture^58^. Poor plant productivity decrease microbial population, together with poor Se bioavailability of northwest China significantly limited Se volatilization. Moreover, soils in northwest China are saline and alkaline, which influences the availability of Se and limited Se volatilization by microorganisms.

**Figure 4:**
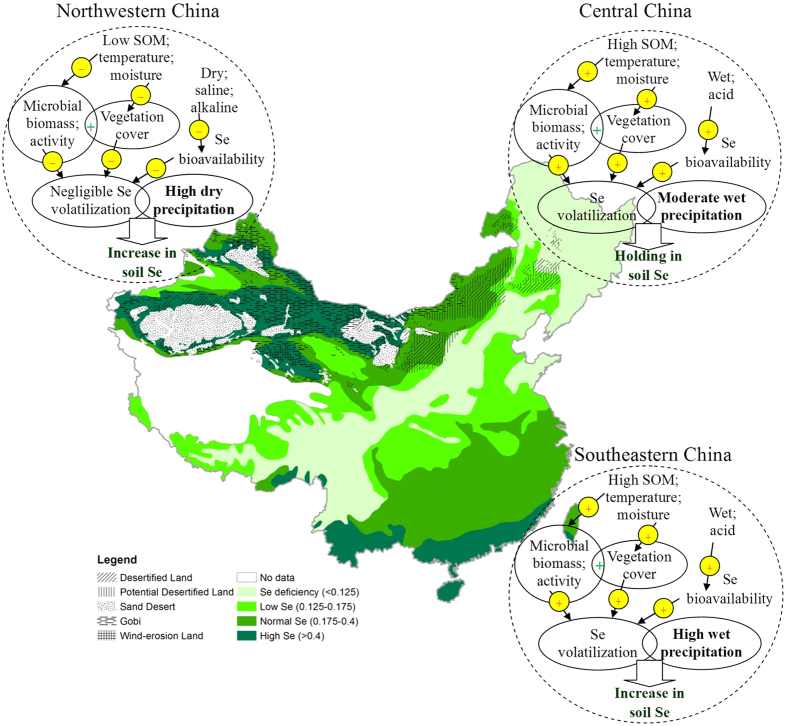
Schematic diagram of major factors and their potential impacts on soil Se volatilization in China, and the effects of volatilization and wet/dry deposition on the distribution of soil Se. SOM refer to soil organic matter. The maps were created using Arc GIS Geographic Information Systems software version 10.2 (Environmental Systems Research Institute Inc, Redlands, Calif).

**Figure 5:**
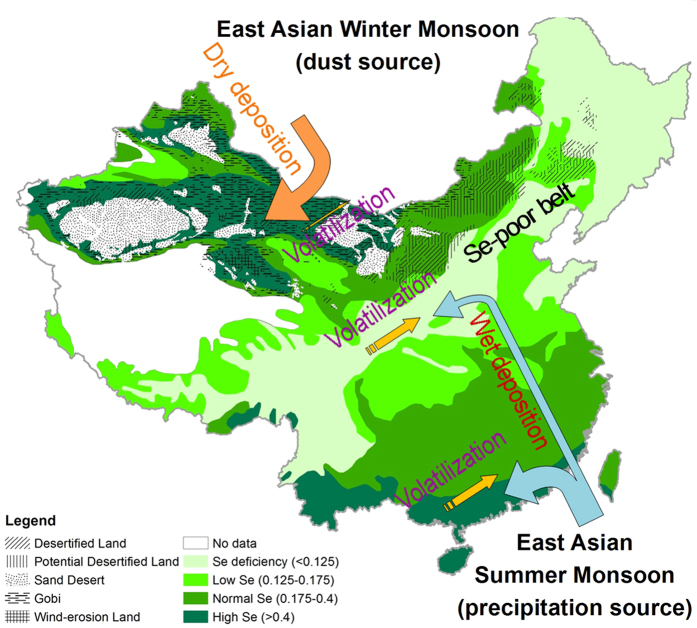
The fluxes of Se deposition and volatilization in three part of territory in China. Southeast humid region (>800 mm); Central dry and humid region (400–800 mm); and Northwestern dry region (<400 mm). The maps were created using Arc GIS Geographic Information Systems software version 10.2 (Environmental Systems Research Institute Inc, Redlands, Calif).

Considering all these factors including low temperature, deposition, moisture availability and vegetation cover, all of which limited microbial biomass, abundances and activity, together with poor Se bioavailability in drier saline-alkaline soils. Selenium volatilization in northwest China would be much less or negligible, especially in the Gobi desert (bare land) (Fig. 4). In desertified and potential desertified lands less Se elevation (normal Se) were observed than Gobi desert (high Se) (Fig. 1A), because these lands were covered by steppe, more Se volatilization would be existed due to higher microorganisms and deposition (200 mm) than that in Gobi desert (<100 mm). It has been demonstrated that the soil microbial biomass carbon in the surface soil in Gobi deserts increased substantially with increasing mean annual deposition^59^,^60^, indicating higher microbial abundance in desertified and potential desertified lands than that in Gobi deserts. It is reasonable to believe that more Se would be volatilized from desertified and potential desertified lands than that in Gobi desert, causing relatively less Se accumulation in surface soil. In a word, high dry deposition from East Asian winter monsoon and negligible Se volatilization predominate the enrichment of Se in soil of Northwest China (Figs 4 and 5).

## Discussion

We hypothesize that spatial variability of soil Se in China is potentially caused by balance between deposition (wet and dry) and volatilization mediated by microorganisms. Absolute accumulation rates were obtained by deducting Se volatilization from total deposition (Table 1).

In southeast China, high Se input occurred from deposition linked with East Asia summer monsoon, especially coastal area (200–400 μg/m^2^/yr). Although soil Se volatilization offset to some degree the Se increase in soil by deposition, high deposition rate substantially improve the Se contents in the soil (Fig. 5 and Table 1). In Central China the rate of Se deposition (40–160 μg/m^2^/yr) was roughly equal to the rate of soil Se volatilization (100–200 μg/m^2^/yr). Much less Se was accumulated in the soil and soil Se is near or a bit higher than that of the bedrock although Se input from monsoon-derived deposition has been for millions of years (Fig. 5). Probably this is the main cause for the formation of Se-poor belt in China. In northwest China it seemed that more Se was volatilized than deposited according to calculation if equal Se volatilization rate in southeast China exist (Table 1) and soil Se in this region should be decreased rather than increased. In fact, Se volatilization is negligible due to the much less microorganisms in dry soil.

In conclusion, we suggest that deposition associated with monsoons (wet and dry) and Se volatilization driven by biology, especially soil microorganisms are the key factors in controlling Se redistribution in terrestrial environments in other regions of the world. Future work should focus on a comprehensive quantification of Se distribution to refine our understanding of the roles of soil microbial communities and climate on Se redistribution and spatial variability not only in China but at the global scale; as such knowledge will provide predictions on Se distribution in arable soils, which will help to prevent health risks related to Se deficiency.

## Methods

When calculating Se wet deposition, we assume that Se concentration in rainfall remains constant. In southeast and central China, wet deposition is the major contributor for soil Se and dry Se deposition is omitted due to unavailable data. In northwest China, dry deposition is the main contributor for surface soil Se and wet deposition is omitted due to much less rainfall. For calculation of Se volatilization, we assume that the rates of Se volatilization in southeast and central China are the same although some factors such as different climate and geology, might affect this value.

## Additional Information

**How to cite this article**: Sun, G.-X. *et al.* Distribution of soil selenium in China is potentially controlled by deposition and volatilization?. *Sci. Rep.* **6**, 20953; doi: 10.1038/srep20953 (2016).

## Figures and Tables

**Figure 2:**
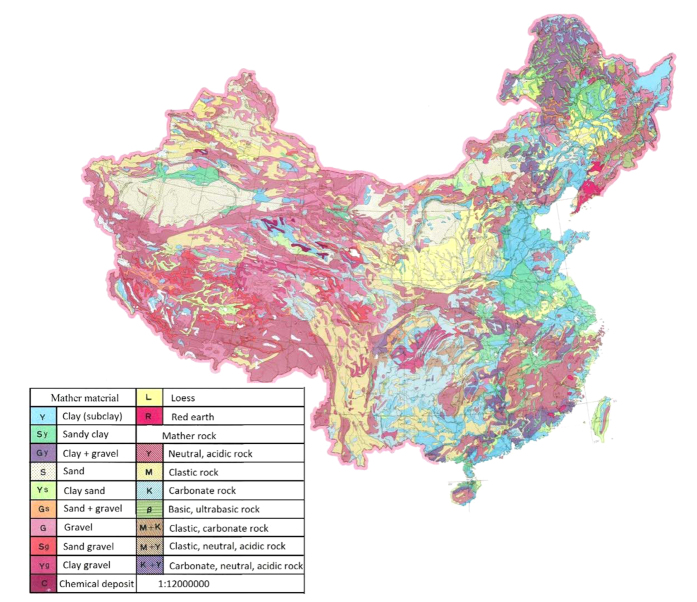
Map of soil parent material and rock of China from a book titled “The atlas of soil environmental background value in the People’s Republic of China”. 1994, page 10–11, China Environmental Science Press. The maps were modified using Arc GIS Geographic Information Systems software version 10.2 (Environmental Systems Research Institute Inc, Redlands, Calif).

**Table 1:** Calculated Se deposition, volatilization and net input (deposition minus volatilization) in soil.

Wet deposition (mm/yr, i.e. L/m^2^/yr)	200	400	800	1600	2000
Se level in rainwater of China (μg/L)	0.1–0.2				
Se deposition (μg/m^2^/yr)	20 ~ 40	40 ~ 80	80 ~ 160	160 ~ 320	200 ~ 400
Average Se volatilization from soil (μg/m^2^/yr)	100–200 (medium 150, grassland)				
Net Se input	−130 ~ −110	−110 ~ −70	−70 ~ 10	10 ~ 170	50 ~ 250
